# A Paleolithic Diet with and without Combined Aerobic and Resistance Exercise Increases Functional Brain Responses and Hippocampal Volume in Subjects with Type 2 Diabetes

**DOI:** 10.3389/fnagi.2017.00391

**Published:** 2017-12-04

**Authors:** Andreas Stomby, Julia Otten, Mats Ryberg, Lars Nyberg, Tommy Olsson, Carl-Johan Boraxbekk

**Affiliations:** ^1^Department for Public Health and Clinical Medicine, Medicine, Umeå University, Umeå, Sweden; ^2^Jönköping County Hospital, Region Jönköping County, Jönköping, Sweden; ^3^Umeå Center for Functional Brain Imaging, Umeå University, Umeå, Sweden; ^4^Department of Integrative Medical Biology, Physiology, Umeå University, Umeå, Sweden; ^5^Radiation Sciences, Diagnostic Radiology, Umeå University, Umeå, Sweden; ^6^Center for Demographic and Aging Research, Umeå University, Umeå, Sweden; ^7^Danish Research Centre for Magnetic Resonance, Centre for Functional and Diagnostic Imaging and Research, Copenhagen University Hospital, Hvidovre, Denmark

**Keywords:** type 2 diabetes, paleolithic diet, exercise, magnetic resonance imaging, episodic memory

## Abstract

Type 2 diabetes is associated with impaired episodic memory functions and increased risk of different dementing disorders. Diet and exercise may potentially reverse these impairments. In this study, sedentary individuals with type 2 diabetes treated by lifestyle ± metformin were randomized to a Paleolithic diet (PD, *n* = 12) with and without high intensity exercise (PDEX, *n* = 12) for 12 weeks. Episodic memory function, associated functional brain responses and hippocampal gray matter volume was measured by magnetic resonance imaging. A matched, but not randomized, non-interventional group was included as a reference (*n* = 6). The PD included a high intake of unsaturated fatty acids and protein, and excluded the intake of dairy products, grains, refined sugar and salt. The exercise intervention consisted of 180 min of supervised aerobic and resistance exercise per week. Both interventions induced a significant weight loss, improved insulin sensitivity and increased peak oxygen uptake without any significant group differences. Furthermore, both interventions were associated with increased functional brain responses within the right anterior hippocampus, right inferior occipital gyrus and increased volume of the right posterior hippocampus. There were no changes in memory performance. We conclude that life-style modification may improve neuronal plasticity in brain areas linked to cognitive function in type 2 diabetes. Putative long-term effects on cognitive functions including decreased risk of dementing disorders await further studies. Clinical trials registration number: Clinicaltrials. gov NCT01513798.

## Introduction

Type 2 diabetes poses a major threat for future health, including a doubled risk of both vascular and neurodegenerative forms of multiple dementing disorders (Biessels et al., 2006). Notably, obesity and type 2 diabetes were recently highlighted as two of the most important modifiable risk factors for development of the most common type of dementia, i.e., Alzheimer's disease (Norton et al., 2014), and targeted interventions to improve cardiometabolic risk factors have been deemed central to lower the incidence of multiple dementing disorders including both Alzheimer's disease and vascular dementia in the future (Biessels et al., 2014).

Longitudinal measurements suggest an increased age-related decline in global cognitive function in subjects with type 2 diabetes (van den Berg et al., 2010; Yaffe et al., 2012; Rawlings et al., 2014). These cognitive deficits including impaired verbal memory function are probably mediated by altered brain structure, such as decreased hippocampal volume and connectivity (Strachan et al., 1997; den Heijer et al., 2003; Awad et al., 2004; Gold et al., 2007; van Bussel et al., 2016), as well as decreased cortical volume and thickness in the prefrontal cortex (Brooks et al., 2013; Brundel et al., 2014). Furthermore, fMRI has revealed impaired functional connectivity of various brain areas, including both the hippocampus, prefrontal and occipital cortices (Xia et al., 2013; Cui et al., 2014).

A paleolithic-type diet has been shown to have major beneficial effects on cardiovascular risk factors in type 2 diabetes (Jönsson et al., 2009). Furthermore, this diet may also improve episodic memory performance, possibly via altered hippocampal function in obese non-diabetic women (Boraxbekk et al., 2015). Notably, aerobic exercise improves memory performance and increases prefrontal cortical volume (Ruscheweyh et al., 2011) and hippocampal volume in individuals without diabetes (Erickson et al., 2011; Jonasson et al., 2016). These effects may be mediated by increased secretion of brain-derived neurotrophic factor (BDNF) (Choi et al., 2009; Erickson et al., 2011). Our aim was therefore to study if an intervention with diet and exercise can improve brain function and structure in individuals with type 2 diabetes.

We hypothesized that a PD would improve episodic memory performance, increase hippocampal volume and increase functional brain responses within the prefrontal cortex and hippocampus during memory encoding. Furthermore, we hypothesized that the addition of aerobic and resistance exercise to the PD would potentiate these effects (Erickson et al., 2011; Ruscheweyh et al., 2011).

## Methods

### Participants

Thirty-two overweight or obese (BMI 25–40 kg/m^2^) individuals with type 2-diabetes (<10 years duration) were recruited in 2012–2014 by advertisements in local newspapers and around the Umeå University Hospital catchment area. Eligible men were 30 to 75 years old and women were postmenopausal up to 75 years old. Exclusion criteria: treatment with insulin, other oral diabetic drugs than metformin, beta-blockers or anti-thrombotic drugs, HbA1c <47 mmol/mol, resting blood pressure >160/100 mmHg, urinary albumin creatinine ratio >30 g/mol, a history of stroke, cardiovascular, psychiatric, kidney, lung, gastrointestinal or liver disease and malignancy, alcohol consumption >16 points (men) or >14 points (women) assessed by the Alcohol Use Disorders Identification Test, major depression (>20 points) assessed by the Montgomery Asberg Depression Rating Scale, allergy to key components of the *PD* > 30 min of moderately intense physical activity more than 5 days per week or resistance exercise more than once every other week during the last 6 months.

Sixteen participants were randomized to either a PD or PD combined with supervised exercise (PDEX) (for details see Otten et al., 2017). The study was single-blinded, such that group allocation was unknown to staff that performed the examinations, dietary counseling and analyzed the data. Three participants did not complete the interventions and 5 participants did not participate in the MRI examinations. Thus, 12 participants from each intervention group were included in the final analysis (Supplementary Figure 1). Based on a previous diet intervention in obese women 18 participants would provide 80% power to detect an improved episodic memory function at *P* < 0.05 (Boraxbekk et al., 2015).

A reference group was included to assess effects of repeated testing on brain imaging and memory performance. These individuals were recruited through advertisements in newspapers and among participants that were declined to participate in the intervention study due to lack of time, treatment with beta-blockers or anti-thrombotic drugs, all other inclusion and exclusion criteria were similar. Nine participants were included, however six participants performed two MRI examinations (2 excluded due to subcutaneous metal, 1 due to scanner problems) and were included in the final analysis.

All participants gave written informed consent before study inclusion. The study protocol was in accordance with the Helsinki declaration and approved by the regional ethical committee of Umeå University.

### Interventions

The PD and PDEX group attained five group sessions separately, in which a dietician instructed them how to eat a PD. Two sessions were held the first 2 weeks after baseline measurements and the following sessions once a month. The PD was consumed *ad libitum* and based on lean meat, fish, eggs, fruits, berries, vegetables, and nuts. It excluded cereals, dairy products, refined fats and sugar and salt. Dietary intake was validated with 4-day weighted food records (for details see Otten et al., 2017).

After the baseline measurements participants in both intervention groups met a physician individually and were motivated to increase their daily physical activity to at least 30 min of moderately vigorous activities. In addition, the PDEX group attained 1-h exercise sessions three times per week. The exercise was performed in groups of 1–4 participants with a professional trainer and consisted of 50 percent aerobic exercise and 50 percent resistance exercise (for details see Otten et al., 2017).

### Clinical measurements

All measurements were performed during a 3-week period before randomization and after 12 weeks of intervention. Weight was measured in light clothing and length on a digital height-measuring gauge. Waist circumference was measured midway between the iliac crest and lower rib during exhalation. Body composition was estimated using dual energy X-ray absorptiometry (GE Medical Systems, Lunar Prodigy X-ray Tube Housing Assembly, Brand BX-1L, Model 8743, Madison, WI, USA).

Fasting serum triglycerides, cholesterol, HDL cholesterol, insulin, HbA1c and blood glucose levels were analyzed with routine clinical laboratory methods at the Department for Clinical Chemistry, Umeå University Hospital. The homeostatic model assessment (HOMA-IR; fasting glucose × fasting insulin/22.5) was used to estimate insulin resistance. LDL cholesterol was calculated as (serum cholesterol—serum HDL—serum triglycerides)/2.2. Fasting serum BDNF levels was analyzed in duplicates with an inter- and intra-assay coefficient of variation <15% (Human Free BDNF Quantikine ELISA Kit, R&D systems, Abingdon, United Kingdom).

Aerobic capacity was estimated by using a standardized cardiopulmonary exercise test and physical activity measured with a combined heart-rate monitor and accelerometer (Actiheart®, CamNtech Ltd., Cambridge, United Kingdom) for 7 consecutive days.

### Episodic memory test

A face-name paired-associates task was used to evoke functional brain responses related to episodic memory encoding and retrieval (Pudas et al., 2013). During encoding the participants memorized faces with associated common Swedish names. Subsequently, during memory retrieval, the same faces were presented with three adjacent letters and the task was to indicate which letter that fit with the first letter in the name associated with the face. They did this by pressing one of three buttons on a scanner compatible response pad, if unsure they were instructed to guess. An active baseline task was performed between the blocks of memory encoding and retrieval; participants pressed a button with their index finger when a circle replaced the fixation cross. The paradigm had a blocked design with 6 blocks of memory encoding and retrieval including 4 faces and names each, and eight blocks of the active baseline task. The median time between memory encoding and retrieval was 85 s. The paradigm was presented with E-prime v.1.1 (Psychology Software Tools, PA, USA) on a computer screen seen through a mirror on the head coil. All participants were right handed.

### MRI acquisition

At baseline and after 12 weeks T2^*^ weighted images were acquired using an echoplanar imaging sequence on a General Electric 3 T Discovery MR 750 scanner with a 32-channel head coil. The parameters were as follows: 37 transaxial slices with a thickness of 3.4 mm and 0.5 mm gap, 2,000 ms repetition time, 30 ms echo time, a flip angle of 80° and a 25 × 25 cm field of view. Ten dummy scans were initiated image collection and later discarded from the analysis to allow for saturation artifacts. For the high resolution T1-weighted structural images a 3D fast spoiled gradient echo sequence with the following parameters was used: 180 transaxial slices, thickness 1 mm, TR 8·2 ms, TE 3·2 ms, flip angle 12°, and field of view 25 × 25 cm.

### Preprocessing and statistical analysis of functional MRI data

SPM12 (Wellcome Department of Imaging Science, Functional Imaging Laboratory, http://www.fil.ion.ucl.ac.uk/spm) implemented in Matlab 2014b (Mathworks Inc., MA, USA) was used to analyze the fMRI data. A program developed in-house (DataZ9D) was used for batching of analyses and extraction of median parameter estimates across clusters. Anatomical images displaying SPMs projected on a group-specific template were generated in MRIcron (http://people.cas.sc.edu/rorden/mricron/index.html). No participant had any sudden head movements >2 mm or rotations >2°, therefore none was discarded due to motion artifacts.

Images were corrected for time-differences in slice acquisition and motion corrected. DARTEL (Ashburner, 2007) was used for realignment of images, normalization to the group-specific template, alignment to the MNI space and smoothing with an eight-millimeter FWHM Gaussian kernel.

The general linear model with each condition (encoding, retrieval and baseline) as separate regressors was used as first-level analyses. Encoding and retrieval were contrasted with the baseline task. In the second level analysis we first performed a one-sample *t*-test including participants in the intervention groups (*n* = 24) to display task specific (encoding and retrieval) SPMs before the intervention (*P* < 0.05, FEW corrected; Supplementary Figure 2). We then applied flexible factorial models to test the effect of time (*n* = 24) and time × group interaction (*n* = 12 + 12) in the PD and PDEX group. The factors subject, group and time were included. The task-specific SPMs were used as explicit masks to restrict the analysis to regions within the encoding and retrieval networks. An uncorrected *P* < 0.001 with a cluster extent >10 contiguous voxels was considered significant in the flexible factorial models.

For the ROI analysis of the hippocampus a hippocampal mask was created from the brain atlas generated by Automated Labeling of Neuroanatomical Structures (Fischl et al., 2002). The same flexible factorial models as specified above were used but with the hippocampal mask as explicit mask. A *P* < 0.001 with a cluster extent >5 voxels was considered significant in this analysis.

For visualization of results and correlation analyses the peak %BOLD signal change in the three intervention groups were extracted from the clusters with a significant time effect or time × group interaction. The %BOLD signal change was calculated with the following formulas ([β_Encoding_ − β_Baseline_]/β_Constant_) × 100 and ([β_Retrieval_ − β_Baseline_]/β_Constant_) × 100, where βs are the regression coefficients from the first level analysis and the β_Constant_ is the mean BOLD-response during the whole face-name paradigm. Since the reference group was small and matched but not randomized this group was not included in the flexible factorial model. Instead, the peak %BOLD signal change in the clusters with a significant time effect or time × group interaction in the intervention groups was compared *post-hoc* between the reference group and the PD and PDEX group using Mann-Whitney *U*-tests.

### Preprocessing and statistical analysis of structural MRI data

A voxel-based morphometric analysis of the hippocampus was performed using the Computational Anatomy Toolbox 12 (http://dbm.neuro.uni-jena.de/cat/). Images were preprocessed and segmented into gray matter and white matter through the longitudinal pipeline using DARTEL (Ashburner, 2007). The preprocessing included an initial realignment of scans at pre- and post-intervention. Thereafter a mean image of these two scans was created for each participant. The mean image was used as a reference for further realignment, correction for signal inhomogeneity, and spatial normalization. Smoothing was done in SPM12 with an 8-mm FWHM Gaussian kernel. The sample homogeneity test was used to identify outliers. One participant in the PDEX group and one participant in the reference group had a low correlation between the baseline and 12 week scan (>2 standard deviations) and were therefore excluded from the structural analysis.

A flexible factorial model was used to test the effect of time and group × time interaction in the intervention groups on gray matter volume within the hippocampus by applying an explicit hippocampus mask (Fischl et al., 2002). A *P* < 0.05 FWE corrected with a cluster extent >5 voxels was considered significant in this analysis. Marsbar v. 0.44 (http://marsbar.sourceforge.net/index.html) was used to extract the mean beta weight, which is an estimate of gray matter volume, from clusters within the hippocampus with a significant time effect or time × group interaction. The beta weights were compared between the reference group and PD and PDEX group using Mann-Whitney *U*-tests.

### Statistical analysis of clinical data

Non-parametric statistical tests were used to test between and within group differences. Spearman correlations was used to test the relationship between changes in memory performance, BMI, body fat percent, fasting insulin levels, fasting glucose levels, HOMA-IR, maximum oxygen uptake and serum BDNF levels with changes in %BOLD signal change and estimated gray matter volume in the hippocampus. A two sided *P* < 0.05 was considered significant in these analyses.

## Results

### Clinical measures and aerobic capacity

Data from the participants who completed the MRI scans are presented. The clinical data from the full sample, including those who did not take part in the MRI scans, has been published previously (Otten et al., 2017). At baseline, the PDEX group had higher fasting blood glucose and HDL cholesterol levels than the PD group (*P* < 0.01 for both). In both the PD and PDEX group there were pronounced reductions in BMI, waist circumference, total body fat, HbA1c, fasting insulin, glucose, HOMA-IR and triglyceride levels (*P* < 0.01 for all; Table 1). Lean mass decreased in the PD group (*P* < 0.01) but was unchanged in the PDEX group, while total cholesterol levels decreased in the PDEX group (*P* < 0.01) but not in the PD group. The peak oxygen uptake increased in both groups without any significant group differences (Group difference in change from baseline, *P* = 0.091). In the reference group, all clinical measures were unchanged from baseline-−12 weeks (Supplementary Table 1). At baseline, the PD group had lower HDL cholesterol levels than the reference group (*P* = 0.04). There were no other baseline differences between the PD and PDEX compared with the reference group.

**Table 1:** Anthropometric, biochemical and fitness measurements [medians (IQR), (range) is given for age].

	**PD**		**PDEX**	
	**Baseline**	**12 weeks**	**Baseline**	**12 weeks**
Gender (Men/Women)	9/3		8/4	
Age (years)	59 (44–66)		61 (58–69)	
Diabetes duration (years)	3 (6)		5.5 (7)	
BMI (kg × m^−2^)	30.6 (4.1)	28.4 (4)^*^	32.1 (7.6)	29.4 (7.4)^*^
Waist (cm)	110 (14)	99 (15)^*^	108 (20)	99 (23)^*^
Total body fat (%)	36.1 (6.5)	31.7 (9.3)^*^	37.7 (9.6)	33.3 (10.4)^*^
Total lean mass (kg)	56.9 (15.9)	55.2 (12.4)^*^	61.0 (24.9)	60.7 (26.6)
HbA1c (mmol/mol)	50 (10)	41 (2)^*^	57 (17)	42 (6)^*^
fP-Insulin (IU)	23 (12)	11.5 (6.5)^*^	16.5 (10.8)	11.0 (5.9)^*^
fB-Glucose	7.5 (1.8)^#^	6.1 (1.2)^*^	9.6 (3.6)^#^	7.2 (1.5)^*^
HOMA-IR	7.2 (3.6)	3.0 (2.5)^*^	7.7 (5)	3.3 (1.6)^*^
fS-Cholesterol (mmol/l)	4.5 (2.0)	4.2 (1.5)	4.3 (1.3)	3.8 (0.9)^*^
fS-LDL (mmol/l)	2.1 (1.0)	2.6 (1.4)	2.3 (1.4)	2.2 (0.8)
fS-HDL (mmol/l)	0.8 (0.2)^#^	0.9 (0.24)	1.1 (0.3)^#^	1.1 (0.22)
fS-Triglycerides (mmol/l)	2.3 (2.8)	1.04 (1.2)^*^	1.7 (1.5)	1.1 (0.4)^*^
fS-BDNF (ng/ml)	26.1 (8.3)	22.8 (8.2)	27.6 (7.3)	21.7 (15.9)
Peak oxygen uptake (ml/min/kg)	23.7 (6.3)	28.2 (9.9)^*^	22.1 (5.0)	26.5 (5.7)^*^

* P < 0.01 for change from baseline—12 weeks within each group tested with Wilcoxon signed ranks test.

# *P < 0.01 for difference between the groups at baseline tested with Mann-Whitney U test. There were no significant group differences in change from baseline—12 weeks*.

### Adherence to diet and exercise intervention

There were no baseline differences in reported energy intake or macronutrient composition between the PD, PDEX and reference group. Both intervention groups reported decreased total energy intake, reduced intake of carbohydrates and saturated fatty acids while the reported intake of protein, mono- and polyunsaturated fatty acids increased (Table 2; *P* < 0.05 for all). The reference group did not report any changes in total energy or macronutrient intake between baseline and 12 weeks (Supplementary Table 2). The measured total physical activity energy expenditure did not differ between baseline and 12 weeks in neither the PD, PDEX (Table 2) or reference group (Supplementary Table 2).

**Table 2:** Reported energy intake, macronutrient composition and physical activity energy expenditure (PAEE) at baseline and 12 weeks [Medians (IQR)].

	**PD**		**PDEX**	
	**Baseline**	**12 weeks**	**Baseline**	**12 weeks**
Total energy intake (kCal)	2,064 (805)	1,446 (880)^*^	1,728 (866)	1,307 (493)^**^
Protein (E%)	17 (4)	24 (4)^**^	19 (4)	26 (8)^**^
Carbohydrate (E%)	40 (10)	26 (14)^*^	39 (16)	25 (5)^**^
Total fat (E%)	38 (5)	43 (10)	35 (14)	46 (12)
Saturated fat (E%)	15 (4)	9 (3)^**^	13 (5)	9 (3)^**^
Mono-unsaturated fat (E%)	14 (3)	22 (8)^*^	14 (5)	23 (8)^*^
Poly-unsaturated fat (E%)	5 (2)	8 (2)^*^	6 (2)	9 (4)^*^
PAEE (kCal/24h)	935 (303)	881 (280)	973 (1,181)	931 (642)

* P < 0.05,

** *P < 0.01 for change from baseline—2 weeks within each group tested with Wilcoxon signed ranks test. There were no significant differences between groups. E%, energy percent*.

### Cognitive performance

There were no significant changes in memory performance. The PD group remembered 17.1 (2.5) of the names at baseline and 17.8 (2.5) after the intervention while the PD-EX group remembered 16.9 (3.3) at baseline and 17.6 (2.9) after the intervention. The reference group remembered 16.2 (4.4) of the names at baseline and 17.2 (2.8) after the intervention.

### Episodic memory network at baseline measurement

At baseline, the face-name paradigm induced significant BOLD responses in a widespread brain network (Supplementary Figure 1). This network included the same brain regions as has been found previously by using this memory paradigm (Kauppi et al., 2011; Salami et al., 2012; Pudas et al., 2013).

### Altered functional brain responses in the intervention groups

**Figure 1:**
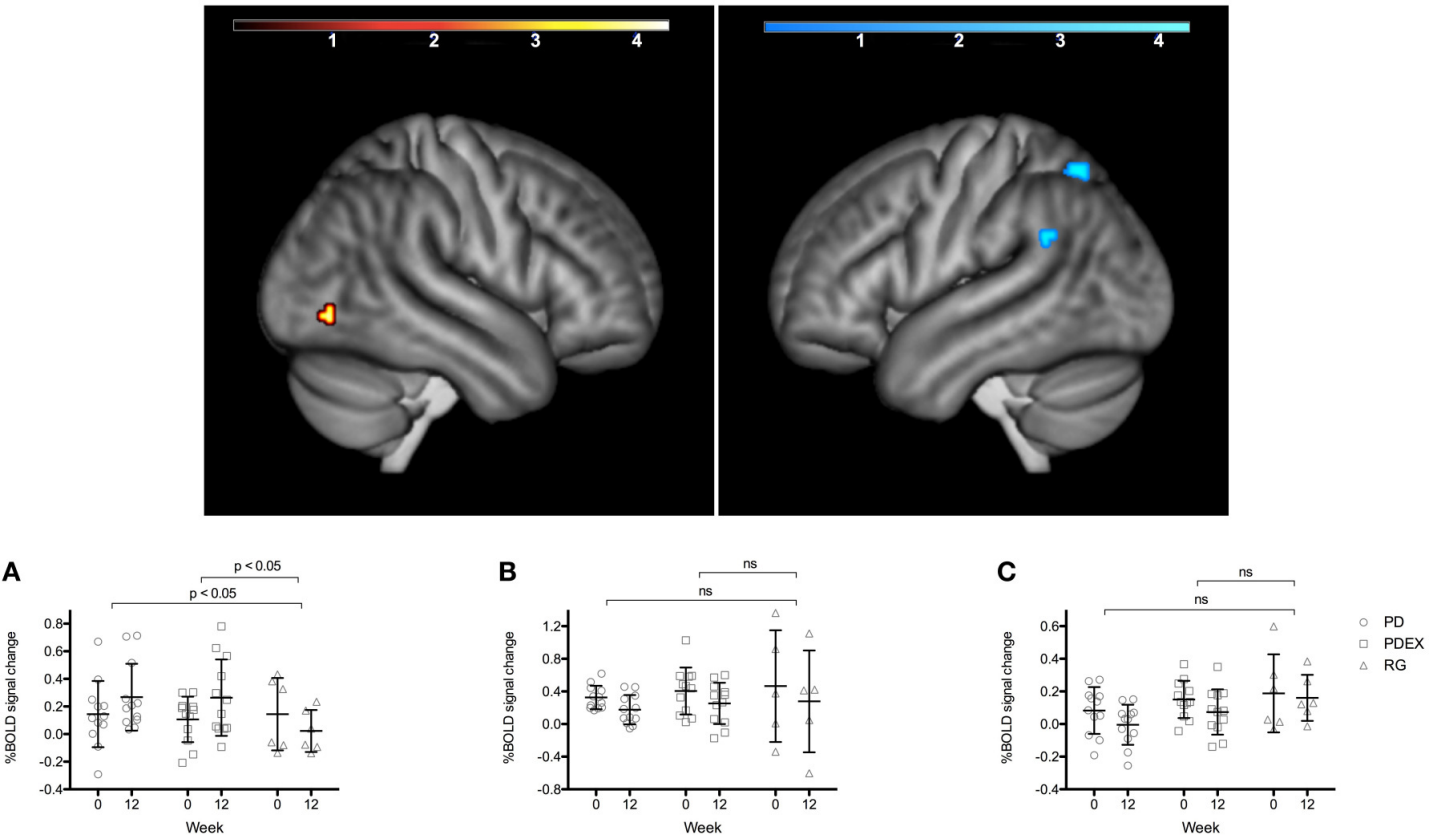
Regions with a significant effect of time during memory encoding in the intervention groups (*P* < 0.001, extension >10 voxels, uncorrected; warm colors indicate clusters with increased BOLD response and cold colors indicate clusters with decreased BOLD response; color scale in *T* scores, range 0.03–4.3). The bar charts display the mean (SEM) %BOLD signal change during memory encoding in **(A)** right inferior occipital cortex, **(B)** left superior parietal gyrus, **(C)** left angular gyrus. Black = PD (*n* = 12), gray = PDEX (*n* = 12), white = reference group (*n* = 6). ns, not significant.

During memory encoding, there was a significant (*P* < 0.001, uncorrected) main effect of time expressed as decreased functional brain responses in the left superior parietal gyrus and the left angular gyrus and increased functional brain responses in the right inferior occipital gyrus in both intervention groups (see Figure 1 and Table 3 for exact locations in MNI space, cluster extent and *T*-values. Individual changes presented in Supplementary Figure 3). During memory retrieval, there were no significant effects of time on functional brain responses. There were no significant group × time interactions on functional brain responses during either memory encoding or retrieval.

**Table 3:** Brain regions with a significant main effect of time (*P* < 0.001, extension > 10 voxels) during memory encoding.

**Brain region**	**BA**	***x***	***y***	***z***	**Volume**	***T***	**Change**
L Superior parietal gyrus	7	−24	−64	56	216	4.36	Decrease
R Inferior occipital gyrus	19	46	−74	−6	184	4.04	Increase
L Angular gyrus	39/48/22/41/40	−48	−50	28	136	4.04	Decrease
R Hippocampus^*^	20	32	−12	−16	248	3.79	Increase

Brain region is given for the peak BOLD response. L, Left; R, Right; BA, Brodmann areas included in cluster. Volume is given in [mm^3^].

* *The cluster in R Hippocampus was found in a region of interest analysis of the hippocampus*.

### Functional brain responses in the hippocampus

**Figure 2:**
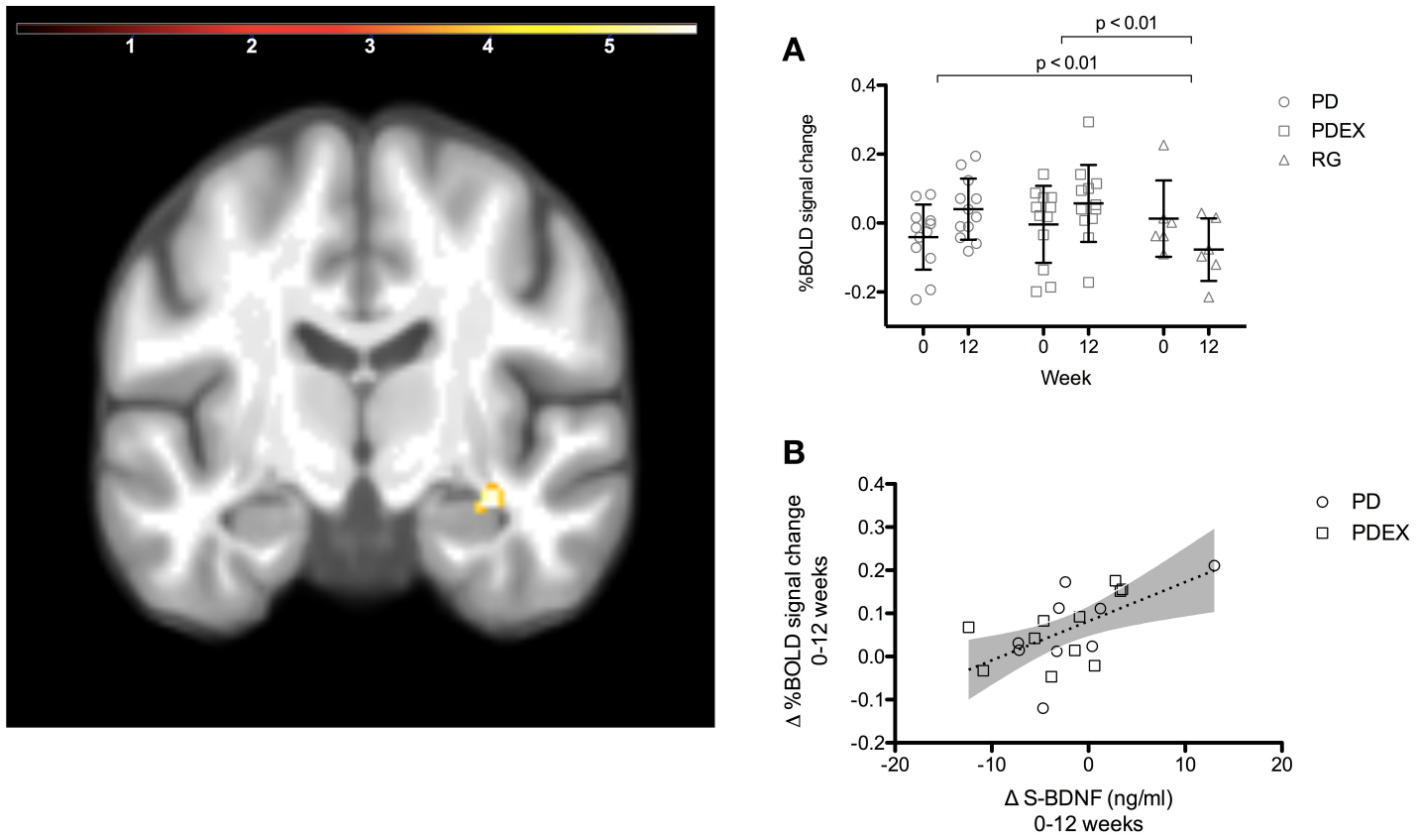
A ROI analysis of the hippocampus revealed increased functional brain response in the right anterior hippocampus in the intervention groups (time effect *P* < 0.001, extension >5 voxels, uncorrected; color scale in *T* scores range 0.03–5.7; *Y* = −12). **(A)** The mean (SEM) peak %BOLD signal change in the cluster at baseline and 12 weeks. Black = PD (*n* = 12), gray = PDEX (*n* = 12), white = reference group (*n* = 6). **(B)** The association between change in serum BDNF levels and %BOLD signal change in the right anterior hippocampus (*r* = 0.58, *P* = 0.007). Dots = PD, Squares = PDEX. Gray areas = 95% confidence interval.

The ROI analysis of the hippocampus revealed a significant (*P* < 0.001, uncorrected) effect of time expressed as increased functional brain responses during memory encoding in the right anterior hippocampus in both intervention groups (Figure 2 and Table 3). This increase over time was significantly different from the decrease in the reference group (Figure 2, PD vs. reference group, *P* = 0.007; PDEX vs. reference group, *P* = 0.003). In the PD group 11/12 participants and in the PDEX 9/12 participants increased the %BOLD response whereas in the reference group 5/6 decreased the %BOLD response (Supplementary Figure 3). There were no significant group × time interactions in the intervention groups.

### Volume of the hippocampus

**Figure 3:**
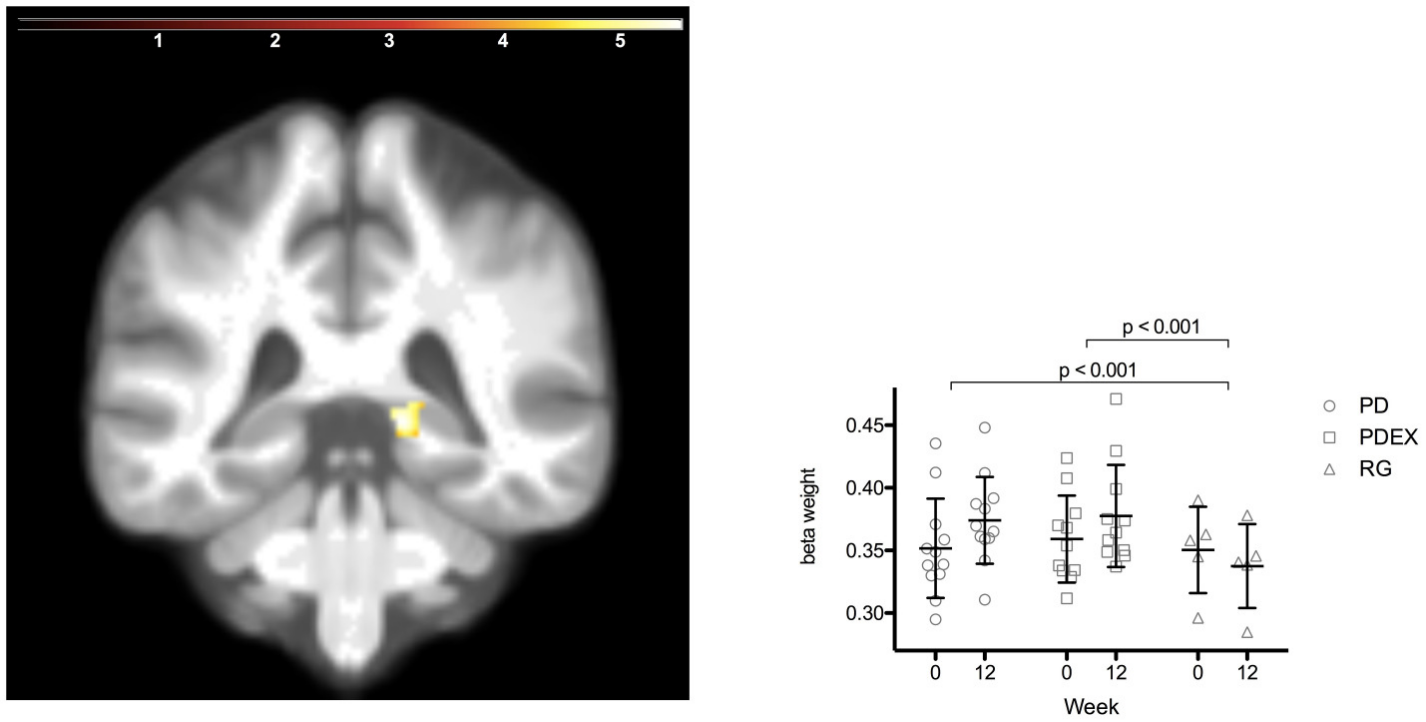
Increased volume in the right posterior hippocampus in the intervention groups (*P* < 0.05 FWE corrected, extension >5 voxels; color scale in *T* scores range 0.03–5.7; *Y* = −38). Bars display the mean (SEM) beta weights in the cluster in right posterior hippocampus. Black = PD (*n* = 12), gray = PDEX (*n* = 11), white = Reference group (*n* = 5).

The ROI analysis of hippocampus structure revealed increased volume in the PD and PDEX group in the right posterior hippocampus [Figure 3; *P* < 0.05, FWE corrected; *T* = 5.8; MNI coordinates (*X* = 15, *Y* = −38, and *Z* = 0); volume 736 mm^3^]. This increase was significantly different from the decrease in the reference group (Figure 3; PD vs. reference group, *P* < 0.001; PDEX vs. reference group, *P* < 0.001). In both the PD and PDEX group 11/12 participants increased the estimated volume whereas it decreased in all participants in the reference group (Supplementary Figure 3). There were no significant group × time interactions on hippocampal volume in the intervention groups.

### Correlations between changes in clinical measures and functional brain responses

In the intervention groups the increased functional brain response in the right hippocampus was associated with increased BDNF levels (*r* = 0.58, *P* = 0.007, Figure 2). Moreover, decreased functional brain response in the left superior parietal gyrus was associated with improved memory performance (*r* = −0.66, *P* < 0.001). There were no significant correlations in the reference group.

## Discussion

To our knowledge, this is the first study evaluating the effects of diet and exercise on functional brain responses during an episodic memory test and hippocampal volume in patients with type 2 diabetes. We found that after weight loss, with associated improved insulin sensitivity and cardiovascular fitness, functional brain responses increased in the right occipital cortex and the right anterior hippocampus compared with a weight-stable reference group. Furthermore, gray matter volume in the right hippocampus increased in the intervention groups.

The hippocampal region is key for encoding of episodic memories (Salami et al., 2012) and hippocampal atrophy is a hallmark of Alzheimer's disease (Jobst et al., 1994). Related to this, type 2 diabetes has been associated with atrophy (den Heijer et al., 2003; Gold et al., 2007) and altered white matter connectivity of the hippocampus (van Bussel et al., 2016). In our study, the estimated volume of the right posterior hippocampus increased 6% in the PD group, 3% in the PDEX group and decreased 4% in the reference group. A previous exercise intervention increased hippocampal volume by about 2% (Thomas et al., 2016) in sedentary middle-aged adults. Notably, we have shown that 6 months of a paleolithic diet can increase functional brain responses in the right hippocampus during memory encoding in obese women without diabetes (Boraxbekk et al., 2015). Moreover, exercise can increase hippocampal volume among elderly obese individuals with associated improvements in spatial memory function (Erickson et al., 2011). Our results strongly suggest that lifestyle interventions can improve both hippocampal structure and function in individuals with type 2 diabetes. This may counteract the increased risk for Alzheimer's disease in this patient group.

The increased functional brain response in the right anterior hippocampus was strongly associated with increased circulating BDNF levels. BDNF is produced within the hippocampus and mediates plasticity such as neurogenesis and synaptic formation in response various stimuli e.g., dietary restriction and exercise (Lee et al., 2002; Marosi and Mattson, 2014). Moreover, exercise interventions in humans have found an association between increased hippocampal volume and increased circulating BDNF levels (Erickson et al., 2011), but others have failed to find an association between hippocampal volume, memory function and BDNF levels (Kim et al., 2015). Notably, in a study performed within our research site the presence of the Val^66^Met allele, known to cause impaired formation and secretion of BDNF (Egan et al., 2003), was associated with reduced functional brain responses in the right hippocampus during memory encoding (Kauppi et al., 2014). Thus, our results suggest that BDNF may be an important factor mediating the positive effects of these interventions on functional brain responses within the hippocampus in patients with type 2 diabetes. However, factors determining the individual response of circulating BDNF to lifestyle interventions should be further studied. Furthermore, although BDNF crosses the blood-brain barrier in rats (Pan et al., 1998), measuring BDNF in cerebrospinal fluid would be of major interest to strengthen these findings.

The reduced functional brain responses in left superior parietal gyrus, which correlated with improved memory performance, may indicate an effect of repeated testing. In contrast, functional brain responses in the right inferior occipital gyrus increased in the intervention groups but decreased in the reference group. Previous studies using resting-state fMRI have found impaired connectivity of the occipital cortex bilaterally in patients with type 2 diabetes, associated with impaired processing speed and memory function (Cui et al., 2014; Chen et al., 2015). Whether the increased encoding-related functional brain response in the right inferior occipital cortex represents an improvement in functional connectivity and thereby a normalized pattern of brain responses remains to be studied.

Both intervention groups were instructed to eat a Paleolithic-type diet. In addition, the PDEX group underwent a structured exercise program including aerobic and resistance training. This resulted in relatively small differences between the groups regarding cardiometabolic factors. The change in hippocampal volume and functional brain responses after the intervention was also similar in both groups. Previous interventions have found that moderately intense exercise is associated with increased hippocampal volume (Erickson et al., 2011). The unexpected lack of additive beneficial effects of more intense exercise on brain plasticity may, at least in part, be explained by increased all day physical activity in the PD group as indicated by the increased oxygen uptake. In addition, the small sample sizes may have limited the potential to find group differences.

Despite major improvements in metabolic regulation and increased functional brain responses in the occipital cortex and right anterior hippocampus, as well as increased volume of the right posterior hippocampus, we did not find an improved episodic memory performance. Previous studies have also failed to find positive effects of diet and exercise, as well as of intensive glucose lowering therapy on cognitive functions among patients with type 2 diabetes (Williamson et al., 2007; Espeland et al., 2014). Moreover, in healthy individuals exercise may improve global memory functions rather than episodic memory specifically (Jonasson et al., 2016). Future studies should therefore include more sensitive tests as well as tests in other cognitive domains and memory systems.

The inclusion of a matched, weight-stable reference group, which provided the ability to test effects of repeated testing is a strength of this study. However, despite being matched to the intervention groups, the reference group was not randomized and consisted of a small number of participants and was therefore not included in the main analysis of the MRI data but rather used as a reference within the brain regions with a significant effect in the intervention groups. Other limitations include the different levels of fasting plasma glucose at baseline, although the level of insulin resistance calculated with HOMA-IR was similar between groups. Furthermore, in this study rather strict exclusion criterion was used to avoid confounding and to be able to perform the exercise intervention without any risk of harming the participants. Therefore, these results need to be corroborated in more long term studies including more participants, aiming to examine whether these alterations in functional brain responses and hippocampal structure can reduce the risk of developing different dementing disorders such as Alzheimer's disease and vascular dementia.

In conclusion, 3 months of a paleolithic-type diet with and without structured exercise, led to increased gray matter volume in the right hippocampus. Furthermore, increased BDNF levels were associated with increased functional brain response within the right hippocampus. This may suggest that life-style modifications can improve hippocampal function in patients with type 2 diabetes and that BDNF may be an important mediator of these effects.

## Author contributions

AS planned and designed the study, included study participants, collected the fMRI data, analyzed the clinical and fMRI data and was responsible for the writing of the publication. JO planned and designed the study, included study participants and analyzed the clinical data. MR planned and designed the study and included study participants. LN designed the fMRI protocol and gave input on the fMRI analysis. TO was the principal investigator of the intervention study, planned and designed the study and included study participants. C-JB planned the fMRI protocol, collected fMRI data and analyzed the fMRI data. All authors contributed to the interpretation of these results and participated in the writing of this publication. AS is the guarantor of this work and takes responsibility for the integrity of the data and the accuracy of the data analysis.

### Conflict of interest statement

The authors declare that the research was conducted in the absence of any commercial or financial relationships that could be construed as a potential conflict of interest.

## Supplementary material

Supplementary Figure 1 CONSORT flow chart displaying the screening, inclusion and exclusion of participants.

## Abbreviations

BDNF: brain derived neurotrophic factor
BOLD: blood-oxygen-level dependent
DARTEL: Diffeomorphic Anatomical Registration using Exponentiated Lie algebra
fMRI: functional magnetic resonance imaging
FWE: family-wise error rate
FWHM: full width at half maximum
MNI: Montreal neurologic institute
PD: Paleolithic-type diet
PDEX: Paleolithic-type diet with 3-h supervised exercise per week
ROI: region of interest
SPM: statistical parametric map.
