# Supplementary figures and images for: A Paleolithic Diet with and without Combined Aerobic and Resistance Exercise Increases Functional Brain Responses and Hippocampal Volume in Subjects with Type 2 Diabetes
Source: Front Aging Neurosci. 2017 Dec 4;9:391. doi: 10.3389/fnagi.2017.00391 (PMC5722796; doi:10.3389/fnagi.2017.00391)

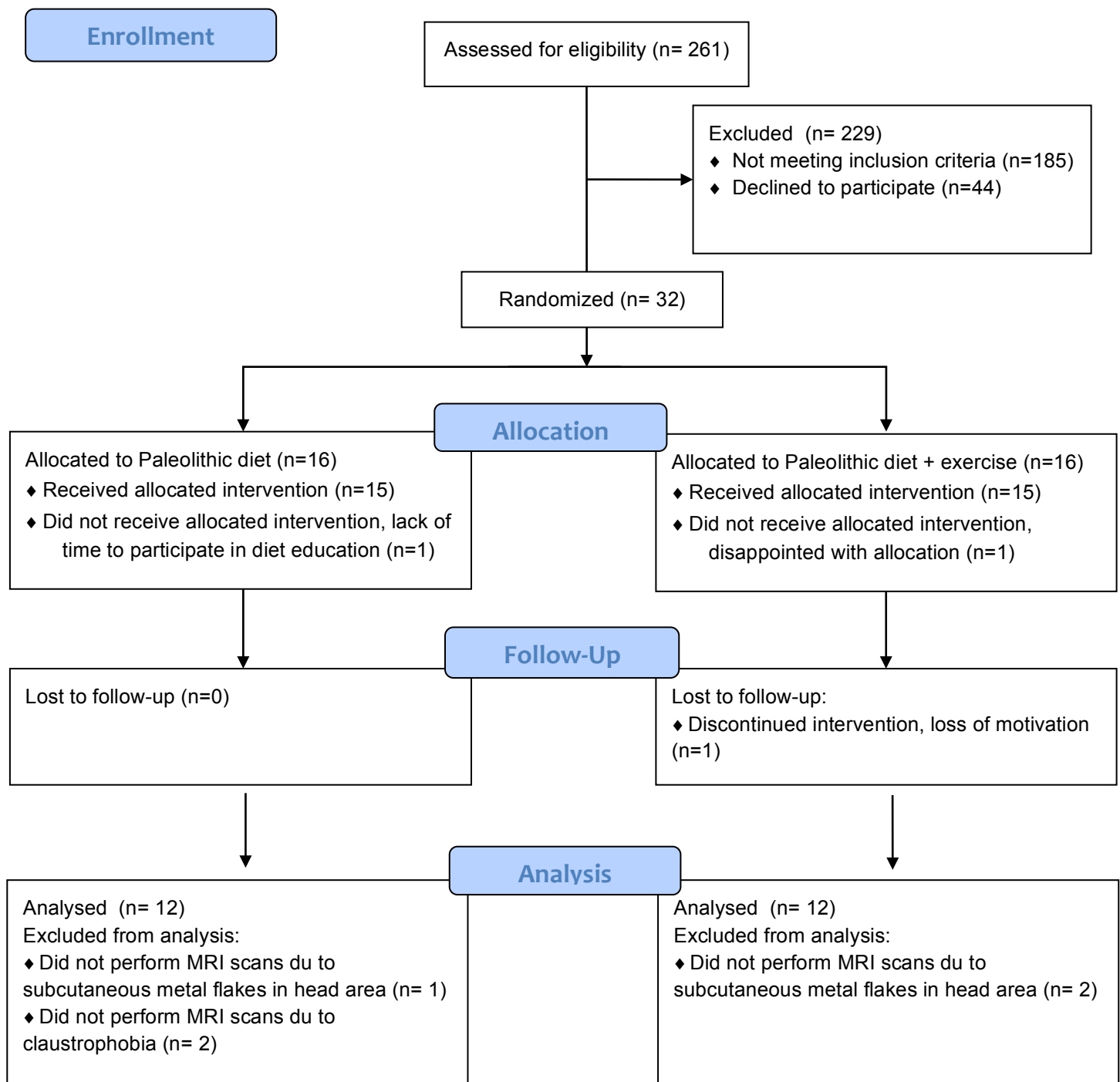

Supplement: Supplementary Figure 1 — CONSORT flow chart displaying the screening, inclusion and exclusion of participants. [file Image1.PDF]
