# Supplementary material for: A Paleolithic Diet with and without Combined Aerobic and Resistance Exercise Increases Functional Brain Responses and Hippocampal Volume in Subjects with Type 2 Diabetes
Source: Front Aging Neurosci. 2017 Dec 4;9:391. doi: 10.3389/fnagi.2017.00391 (PMC5722796; doi:10.3389/fnagi.2017.00391)
Supplement: Supplementary file 2 [file Image2.PDF]

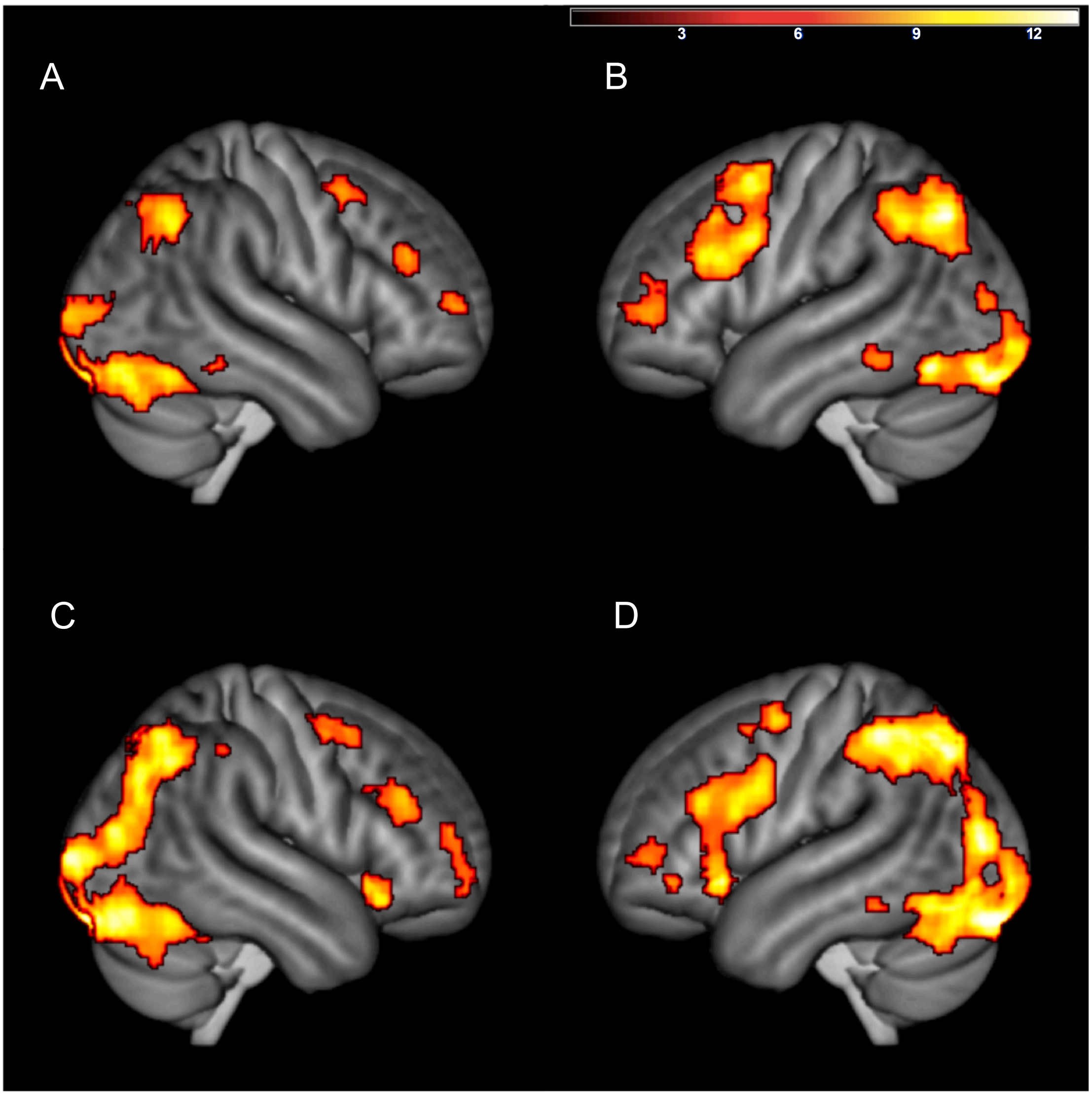

Supplementary figure 2. Statistical parametric maps presented on a group-specific template displaying functional brain responses associated with episodic memory encoding (A-B) and retrieval (C-D) in the intervention groups (n = 24) at baseline. Color scale in *T* scores (range 0.1-13). $P < 0.05$ corrected for the family-wise error rate.
