# Supplementary material for: A Paleolithic Diet with and without Combined Aerobic and Resistance Exercise Increases Functional Brain Responses and Hippocampal Volume in Subjects with Type 2 Diabetes
Source: Front Aging Neurosci. 2017 Dec 4;9:391. doi: 10.3389/fnagi.2017.00391 (PMC5722796; doi:10.3389/fnagi.2017.00391)
Supplement: Supplementary file 3 [file Image3.PDF]

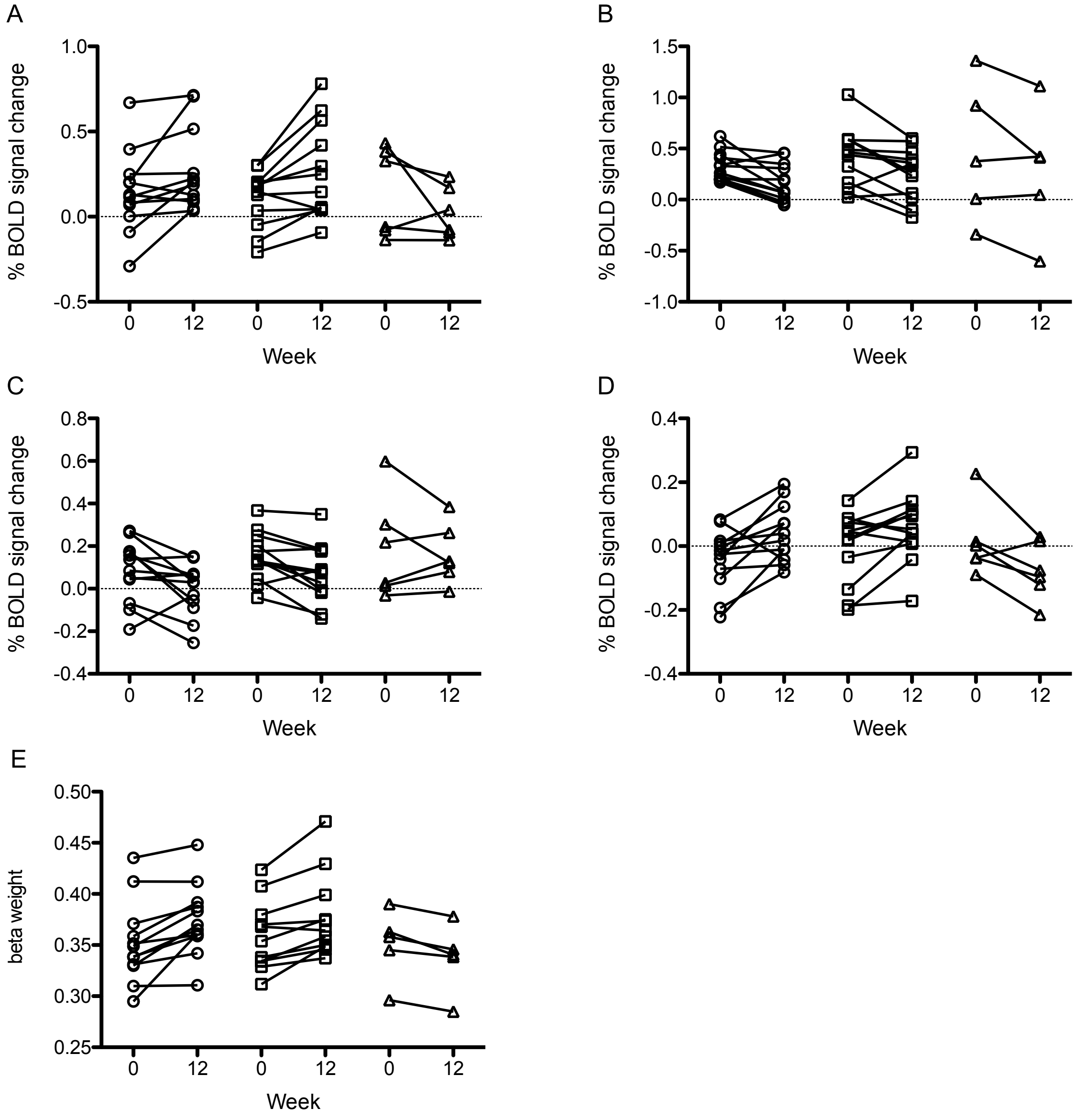

Supplementary figure 3. The individual %BOLD signal change before and after the intervention in the same clusters as in figure 1 and 2. A) right inferior occipital cortex, B) left superior parietal gyrus, C) left angular gyrus, D) right anterior hippocampus. E) Represents the individual beta weights from the voxel based morphometric analysis of hippocampal grey matter volume in right posterior hippocampus, same cluster as in figure 3. Circles = PD (n=12), squares = PDEX (n=12 in A-D and 11 in E), triangles = reference group (n=6 in A-D and 5 in E).
