# Supplementary material for: A Paleolithic Diet with and without Combined Aerobic and Resistance Exercise Increases Functional Brain Responses and Hippocampal Volume in Subjects with Type 2 Diabetes
Source: Front Aging Neurosci. 2017 Dec 4;9:391. doi: 10.3389/fnagi.2017.00391 (PMC5722796; doi:10.3389/fnagi.2017.00391)
Supplement: Supplementary file 4 [file Table1.DOCX]

|  | Reference group | |
| --- | --- | --- |
|  | Baseline | 12 weeks |
| Gender (Men/Women) | 4/2 |  |
| Age (years) | 65 (55-70) |  |
| Diabetes duration (years) | 3.5 (4) |  |
| BMI (kg x m^-2^) | 32.4 (4.3) | 32.5 (4) |
| Waist (cm) | 114 (12) | 115 (11) |
| Total body fat (%) | 40.0 (8.9) | 38.2 (10.5) |
| Total lean mass (kg) | 55.7 (18.6) | 57.6 (21.3) |
| HbA1c (mmol/mol) | 55 (14) | 49 (12) |
| fP - Insulin (IU) | 17.0 (4.8) | 17.0 (12.1) |
| fB - Glucose | 7.3 (1.3) | 7.7 (1.5) |
| HOMA-IR | 5.1 (3.3) | 6.1 (6.7) |
| fS - Cholesterol (mmol/l) | 4.5 (1.8) | 4.4 (1.0) |
| fS - LDL (mmol/l) | 2.3 (2.0) | 2.4 (0.8) |
| fS - HDL (mmol/l) | 1.3 (0.8) | 1.3 (0.7) |
| fS - Triglycerides (mmol/l) | 2.4 (1.2) | 1.7 (0.7) |
| fS - BDNF (ng/ml) | 18.6 (12.4) | 21.7 (14.7) |

**Supplementary table 1.** Anthropometric and biochemical measures in the reference group at baseline and after 12 weeks [medians (IQR), (range) is given for for age]. There were no significant changes over time.
