# Supplementary material for: A Paleolithic Diet with and without Combined Aerobic and Resistance Exercise Increases Functional Brain Responses and Hippocampal Volume in Subjects with Type 2 Diabetes
Source: Front Aging Neurosci. 2017 Dec 4;9:391. doi: 10.3389/fnagi.2017.00391 (PMC5722796; doi:10.3389/fnagi.2017.00391)
Supplement: Supplementary file 5 [file Table2.DOCX]

|  | Reference group | |
| --- | --- | --- |
|  | Baseline | 12 weeks |
| Total energy intake (kCal) | 1731 (587) | 1857 (591) |
| Protein (E%) | 19 (8) | 15 (9) |
| Carbohydrate (E%) | 42 (12) | 40 (17) |
| Total fat (E%) | 37 (5) | 35 (13) |
| Saturated fat (E%) | 11 (6) | 13 (6) |
| Mono-unsaturated fat (E%) | 14 (3) | 14 (5) |
| Poly-unsaturated fat (E%) | 7 (3) | 6 (3) |
| PAEE (kCal/24h) | 800 (481) | 724 (552) |

**Supplementary table 2.** Reported energy intake, macronutrient composition and physical activity energy expenditure (PAEE) at baseline and 12 weeks. [Medians (IQR)].
